# Avoiding the Temperature Danger Zone: A Slovenian Consumer Food Safety Study on Knowledge, Attitudes, and Practices Based on Questionnaire Analysis

**DOI:** 10.3390/foods15061062

**Published:** 2026-03-18

**Authors:** Maja Bensa, Mojca Jevšnik Podlesnik, Lato Pezo, Irena Vovk

**Affiliations:** 1 Research Institute of Faculty of Health Sciences, Faculty of Health Sciences, University of Ljubljana, Zdravstvena pot 5, 1000 Ljubljana, Slovenia; 2 Department of Sanitary Engineering, Faculty of Health Sciences, University of Ljubljana, Zdravstvena pot 5, 1000 Ljubljana, Slovenia; 3 Institute of General and Physical Chemistry, University of Belgrade, Studentski trg 12/V, 11000 Belgrade, Serbia; latopezo@yahoo.co.uk; 4 Laboratory for Food Chemistry, National Institute of Chemistry, Hajdrihova 19, 1000 Ljubljana, Slovenia; irena.vovk@ki.si

**Keywords:** food safety knowledge, attitudes and practices, thawing, heat treatment of food, keeping hot food hot, leftovers, food safety culture, structural equation modeling

## Abstract

There is no doubt that food safety is important for public health and also no doubt stakeholders from farm to fork, including consumers, need to ensure that food remains safe. In Europe, foodborne outbreaks often occur in consumers’ homes, highlighting the importance of research on consumer food safety that leads to interventions. This article reports findings from a part of the Consumer Food Safety Study in Slovenia on the topics of thawing, heat treatment of food, keeping hot food hot and leftovers. A validated online questionnaire was designed using the Matrix of Consumer Food Safety and was completed by 1621 adults. The study assessed consumer food safety knowledge, attitudes and food-handling practices using descriptive statistics, and analyzed how knowledge, attitudes, and practices are interconnected using structural equation modeling. For the most part, participants showed good knowledge, positive attitudes and safe practices, but improvements are needed on thawing methods, use of kitchen thermometers, keeping heat-treated food hot at above 63 °C, and safe cooling and labeling of leftovers. Structural equation modeling in a variety of ways found that (1) knowledge affects attitudes, (2) knowledge affects practices, and (3) attitudes affect practices—emphasizing the importance of including all three aspects in public health food safety interventions. This study offers useful insights and directions for future research and development of public health programs.

## 1. Introduction

Consumer food safety is important because it protects public health and can even save lives, especially for those who are more vulnerable and who find it more difficult to recover from foodborne illness. In addition, food safety is connected with supporting nutrition as foodborne infections can lead to malnutrition, especially in young children. The main challenge is to prevent foodborne diseases, which affect people every day around the world, who get sick because of eating food contaminated with pathogenic microorganisms and/or toxic chemicals [1]. Fortunately, most foodborne diseases can be prevented with proper food handling [1]. A large share of foodborne outbreaks in the European Union occur in consumers’ homes [2]. Consumers at home must rely on themselves to maintain their food safety, whereas other stakeholders in the food safety chain are guided by regulations, inspections, etc. Therefore, it is important—even imperative—that public health institutions learn about problematic areas from consumer food safety studies, such as this one, and address the problems through consumer education and awareness campaigns. Studies on food safety topics report that some consumers are successfully managing, while other consumers could benefit a lot from enhanced knowledge, attitudes and practices.

During food preparation, consumers often engage in thawing, heat treatment of food and sometimes storing leftovers. These activities can help maintain food safety but can also have a negative impact on food safety if not done properly. The major factor for food safety during these food-handling activities is to avoid having food in the so called “danger zone”, which refers to temperature ranges of about 8–63 °C [3] or 5–60 °C [4] or 4.4–60 °C (40–140 °F) [5], in which bacteria may grow more rapidly. Different food safety authorities use different temperature ranges not just because of different temperature units (°C and °F), but also because of different approaches to food safety. For example, European food standards were described as more precautionary and the USA standards as more risk-based [6].

Although there are multiple safe thawing methods that ensure consumers avoid the temperature danger zone, studies reported about consumer knowledge on both safe [7,8] and unsafe thawing methods [9,10,11,12,13,14]. Most of the studies investigated consumer thawing practices, which included the use of the refrigerator [13,15,16,17], microwave [10,11,18,19], and water (in a bowl, in a sink, or under running water) [17,20,21,22]. Unfortunately, the unsafe practice of leaving food to thaw at room temperature [7,15,23,24], or to thaw on the counter [19,20,21,25], or in the kitchen sink or on the bench [26] was used by consumers in different countries. Some consumers skip thawing and opt to cook food from frozen [11,15,19,22].

Proper heat treatment during cooking and reheating destroys pathogens and ensures food is safe for consumption. Studies on consumer knowledge about thorough cooking examined understanding of the importance of thorough cooking for food safety [14,27,28,29]. One study focused on consumer attitudes towards sufficient heat treatment [30]. Other studies reported on consumer use of safe (thermometers) [7,31,32,33] and unsafe (subjective such as smell, taste, and color) [12,13,19,33] methods for determining whether food is sufficiently cooked when heat treating. Studies also investigated consumer knowledge [34,35,36,37] and practices [10,18,25] regarding keeping heat-treated food hot.

Challenges during leftover management include cooling food and refrigerating or freezing as soon as possible—again to avoid the temperature danger zone. There are different approaches to safely cooling and storing leftovers, which also need to be properly labeled (name and date). Thorough reheating is recommended before consumption of leftover foods. Consumer practices for storing leftovers varied in terms of how quickly and in what location food was cooled down as well as how quickly and where leftovers were stored [9,36,38,39,40]. Studies covered different aspects of consumers’ leftover food handling before consumption, including whether reheating was performed [11,14,21,41], where reheating was done [22,39], and how long the reheating took [24,34,42,43]. Consumers also used their leftovers across different time frames [39,41,44].

Studies of consumer food safety from different countries used diverse methods to investigate knowledge (K), attitudes (A), and practices (P)—KAP. Consumers’ knowledge about food safety, their attitudes toward safe food handling, and their everyday food-handling practices shape consumer food safety culture. In the Central European region, there is limited information available about consumer food safety. A few studies previously conducted in Slovenia lacked a comprehensive approach and did not address all three KAP factors across different food-handling activities. Some of these studies included smaller sample sizes or specific population groups. The Consumer Food Safety Study (CFSS) was performed to address these gaps through a mixed-methods investigation of consumer food safety KAP among adults in Slovenia. The CFSS included several topical sections, among which “Food shopping, transportation and refrigeration”, “Food labeling and food preparation” [45], as well as “Food safety information, education and experience”, “Washing hands and cleaning the kitchen”, and “Pets” [46] were previously reported. The novelty of the CFSS lies in its comprehensive investigation of consumer food safety across all three KAP components and a wide range of consumer food-handling topics using a questionnaire developed based on the novel Matrix of Consumer Food Safety. Furthermore, the study included a large sample of consumers in Slovenia and applied structural equation modeling to examine interrelationships among KAP domains. To the best of our knowledge, structural equation modeling has not previously been used in consumer food safety research in Slovenia. This article—a part of the CFSS—aims to present: 1. levels of KAP and 2. structural equation modeling (SEM) analysis of interrelationships among KAP aspects for the topical section “Thawing, heat treatment of food and leftovers”.

## 2. Materials and Methods

### 2.1. The Consumer Food Safety Study and Questionnaire Design

The Consumer Food Safety Study (CFSS) received approval from the University of Ljubljana Biotechnical Faculty Ethical Committee (“Komisija za etično presojo raziskav s področja prehrane (KEP)—KEP-3-12/2023”). The CFSS had two aims: 1. to assess the levels of food safety-related knowledge, attitudes, and food-handling practices among Slovenian adults, and 2. to analyze how knowledge affects attitudes and practices, as well as how attitudes affect food-handling practices.

The CFSS was carried out using a validated questionnaire. Research questions for the questionnaire were designed using the Matrix of Consumer Food Safety (MCFS), a framework established to assess consumer knowledge, attitudes, and practices (KAP) across various food safety topics. This novel, systematic questionnaire design ensured that the questions effectively covered both KAP and the important food safety topics. Development of the MCFS followed several stages: 1. identifying major factors influencing food safety, 2. mapping critical points in consumer food handling, and 3. formulating questions for investigating KAP for each of the food safety topics. The part of MCFS relevant to the questions examined in this article is provided in Appendix A.

The CFSS questionnaire was written in the Slovene language and comprised 60 compulsory questions. The questionnaire included several question formats such as Likert scales (1–5), multiple-choice questions, and open-ended responses. Depending on the context of the Likert scales, the value of 1 denoted “strongly disagree”, “not important at all”, or “never” and the value of 5 corresponded to “strongly agree”, “very important”, or “always”, with the intermediate values 2–4 representing gradual levels in between. This scale was consistently applied throughout the questionnaire, enabling participants to express varying levels of agreement, importance, or frequency.

The CFSS questionnaire was structured into seven topical sections: 1. Demographic, 2. Food safety information, education and experience; 3. Food shopping, transportation and refrigeration; 4. Washing hands and cleaning the kitchen; 5. Food labeling and food preparation; 6. Thawing, heat treatment of food and leftovers; and 7. Pets. The Demographic section collected information on 1. gender, 2. age, 3. education (last completed level of schooling) and 4. household members who are more vulnerable to foodborne disease. Topical sections 3 (Food shopping, transportation, and refrigeration) and 5 (Food labeling and food preparation), which also reported on the pilot testing and validation of the questionnaire, were discussed in a previous article [45]. Topical sections 2 (Food safety information, education and experience), 4 (Washing hands and cleaning the kitchen), and 7 (Pets) were previously presented [46]. This article covers the topical section 6 “Thawing, heat treatment of food and leftovers”. The reliability analysis of section 6 of the pilot questionnaire “Thawing, heat treatment of food and leftovers” (28 items: K29-K31, A15-A18, and P79-P99) yielded a Cronbach’s alpha of 0.878, indicating strong internal consistency.

### 2.2. Questionnaire Administration

The CFSS questionnaire was hosted on the online survey platform 1KA, developed by the University of Ljubljana Faculty of Social Sciences (https://www.1ka.si, accessed on 1 December 2025 [47]). Data collection took place from October 2023 to May 2024. The target audience were consumers in Slovenia who were older than 18 years. Participants were recruited through snowball sampling by disseminating information about the survey via email, flyers, and posters shared across major public institutions (e.g., libraries, universities, research institutes, and secondary schools) as well as state institutions (e.g., ministries) throughout Slovenia. Recruitment of participants was also conducted through social media channels, including Facebook, LinkedIn, Instagram, and X/Twitter. Participants were required to read the “Informed consent to participate in the study” and to indicate their agreement by ticking a confirmation box before proceeding to the first question.

### 2.3. Statistical Analysis

Normality was evaluated using the Shapiro–Wilk and Anderson–Darling tests, which indicated significant departures from normality for Likert-type variables. Given the ordinal nature of the data and the violation of normality assumptions, nonparametric statistical methods were employed. Group differences were examined using the Kruskal–Wallis test. All statistical analyses were conducted using IBM SPSS Statistics (Version 26). Descriptive statistics were used to summarize CFSS participants’ knowledge, attitudes, and practices (KAP) levels.

Structural equation modeling (SEM) was performed using IBM AMOS (Version 21) to test the hypothesized KAP models. As an extension of multiple regression analysis, SEM enables the simultaneous estimation of measurement models and structural relationships among latent constructs, thereby providing a more robust analytical framework than conventional regression approaches. Latent variables were specified according to the conceptual domains of the questionnaire, while observed variables corresponded to individual survey items.

Model fit was assessed using multiple goodness-of-fit indices, including the Root Mean Square Residual (RMR), Goodness of Fit Index (GFI), Adjusted Goodness of Fit Index (AGFI), Normed Fit Index (NFI), Tucker–Lewis Index (TLI), Incremental Fit Index (IFI), Comparative Fit Index (CFI), Parsimony Normed Fit Index (PNFI), Parsimony Comparative Fit Index (PCFI), and the Root Mean Square Error of Approximation (RMSEA), along with its 90% confidence interval and the PCLOSE statistic. These indices were used to evaluate absolute model fit, incremental fit, and parsimony-adjusted fit. Missing data were handled using listwise deletion, and all analyses were conducted on complete cases only.

## 3. Results and Discussion

### 3.1. Demographic Characteristics of Participants

The CFSS questionnaire was completed by 1621 participants, of whom 78.9% (1279) were women, 20.9% (338) men, and 0.2% (4) identified as other [45]. Participants ranged in age from 18 to 90 years, with the following age distribution: 21.96% were in their 20s (18–29 years), 15.18% in their 30s, 22.39% in their 40s, 24.68% in their 50s, 11.10% in their 60s, and 4.69% were aged from 70 to 90 [45]. Most participants (82.97%; 1345) had tertiary education (university or higher education), while 16.53% (268) completed secondary school and 0.49% (8) had primary school or lower [45]. Some participants reported living with household members who are at higher risk for foodborne illness: 12.1% (196) had children under six years of age, 20.9% (339) lived with persons older than 65 years, 23.9% (388) with persons with chronic diseases, and 3.5% (57) with pregnant or breast-feeding mothers [45].

### 3.2. Levels of Knowledge (K), Attitudes (A) and Practices (P)—KAP

#### 3.2.1. Thawing (Defrosting)

The vast majority of CFSS participants agreed (49%) or strongly agreed (40%) that proper thawing of frozen food ensures food safety (Table 1). Other studies noted that rather small numbers of consumers had **knowledge about unsafe thawing** of food at room temperature. In Poland, 37% of consumers knew that thawing food at room temperature represents a high risk of food poisoning [9]. Similarly, in Lebanon, 28% of consumers knew frozen food should not be thawed at room temperature [10]. In the Republic of Korea, many consumers believed that thawing frozen foods at room temperature is hazardous (2010: 26% completely and 37% mostly and 2019: 17% completely and 31% mostly) [11]. In the following countries, less than 20% of consumers knew that thawing food at room temperature is unsafe: Thailand (19%—thawing food at room temperature represents a high risk of food poisoning) [9], Malaysia (16%—knew frozen food should not be thawed at room temperature) [12], Egypt (13%—knew the riskiest way to defrost frozen meat is on the kitchen counter) [13], and Bangladesh (6%—knew that the least safe way to defrost is on the chopping board) [14]. Studies also described consumer **knowledge about safe thawing** methods in the USA (most older adults—in the refrigerator, microwave or cold water) [7], and Bosnia and Herzegovina (67%—on the lowest refrigerator shelf) [8].

CFSS participants had diverse **attitudes** about the importance of the way of thawing frozen food as this was very important for 21%, important for 51%, neither important nor unimportant for 21%, and unimportant for 4% (Table 2).

CFSS participants had diverse **practices** for thawing frozen food and also used more than one approach. The most common approach used by 65% of participants was to thaw food on the counter at room temperature (Table 3). More than half of participants (53%) thaw food in the refrigerator and around one quarter of the participants use the microwave (26%) or cold water (25%) for thawing (Table 3). Thawing food on the counter is not recommended because the outer layers quickly enter the temperature danger zone while the inside is still frozen, increasing both microbial growth and the risk of cross-contamination from dripping thawing liquids. Thawing in the refrigerator allows food to be at a safe constant temperature [48]. Thawing in a bowl of cold water can be faster than refrigerator thawing but attention should be paid to the duration of thawing [48]. Diverse thawing practices described in other studies can be grouped into four categories: 1. in the refrigerator, 2. in/under water, 3. in the microwave, and 4. cooking frozen without thawing.

Thawing in the **refrigerator** was reported for consumers in the following countries: Bosnia and Herzegovina (50%—on the lowest refrigerator shelf) [8], Ireland (49%) [15], the U.S. Virgin Islands (39%) [19], Türkiye (41%—frozen fish) [25], Jordan (37%) [16], Egypt (36%) [13], the USA (33% of parents of elementary school children from Texas) [20], Serbia (27–29% of university students) [18], Slovenia (52% [49], 25% [37], and 17% [22]), the United Arab Emirates (24% of women—frozen meat or poultry) [42], the Republic of Korea (17% in 2010 and 23% in 2019—place in the refrigerator for 1–2 days before it is cooked) [11], Poland (17%) [21], Lebanon (17%) [26], and Ghana (<1%—frozen chicken) [17]. Using the **microwave** for thawing was less frequent but still done by consumers in the Republic of Korea (25% in 2010 and 16% in 2019) [11], Serbia (13–19% university students—meat/chicken) [18], Slovenia (13%—poultry [37] and 11%—meat [22]), Lebanon (13% [10] and 2% [26]), Poland (9%) [21], Ghana (6%—chicken) [17], and the U.S. Virgin Islands (3%—beef) [19].

Thawing frozen food **with water** in a bowl, in a sink, or under running water was also done by consumers in Ghana (40% in a bowl of water—chicken) [17], the USA (the parents of elementary school children from Texas: 39% in water in the sink and 21% under running water) [20], Türkiye (36% in water—fish) [25], Saudi Arabia (women: 35% in warm or hot water—meat) [24], Lebanon (25% under running water) [26], Poland (24% in warm water) [21], Slovenia (24% under tap water—poultry [37] and 13% in hot water—meat [22]), the U.S. Virgin Islands (in the sink with 20% warm water or 16% cold water and 1% bowl with water) [19], Serbia (14–20% of university students: under running water for 1 h—meat/chicken) [18], the Republic of Korea (17% in 2010 and 15% in 2019—dip in water at room temperature) [11], and Ireland (10% in water in the sink) [15].

The unsafe practice of leaving food to thaw **at room temperature** was common for consumers in several countries. In Slovenia, 77% of women (81% nonpregnant and 73% pregnant and postpartum) most frequently thawed raw foods on the kitchen counter [23], 60% of consumers thawed poultry on the kitchen surfaces at room temperature [37], and 50% of consumers [22] as well as 43% of elderly consumers [43] thawed meat on the kitchen counter. Thawing at room temperature was also reported for consumers in Saudi Arabia (54% of women: meat) [24], Poland (22%) [9], Ireland (39%—chicken left overnight) [15], the Republic of Korea (37% in 2010 and 44% in 2019—on the counter) [11], the USA (less than a quarter of older adults: poultry [7]), Italy (7%—fish products for an unspecified time) [50], and Thailand (4%) [9]. Thawing on the counter without specifying room temperature was reported for consumers in Poland (45%) [21], Türkiye (36%—fish) [25], Ghana (35%—chicken) [17], Serbia (31–39% university students: meat/chicken for 1 h) [18], the U.S. Virgin Islands (24%—beef) [19], and the USA (7% of parents of elementary school children from Texas) [20]. In Lebanon, 36% of consumers left frozen food in the kitchen sink or on the bench [26].

A smaller number of consumers opted to **cook frozen** food without thawing in Lebanon (19%) [26], Slovenia (6%—meat) [22], the U.S. Virgin Islands (4% beef) [19], the Republic of Korea (4% in 2010 and 3% in 2019) [11], Ireland (2%—chicken) [15], and Ghana (1%—chicken) [17]. Some studies combined consumers’ answers regarding thawing practices; for example, in New Zealand (34%—bottom shelf in the refrigerator or under running water in the sink—frozen raw poultry) [51], and Saudi Arabia (12% of women—the refrigerator or the microwave) [24]. Other studies highlighted whether consumers’ thawing practices were safe (in Lebanon 68% [32], 64% [26], and 42% [30]) or unsafe (in Egypt 64% [13]).

#### 3.2.2. Sufficient Heat Treatment of Food

CFSS participants had good **knowledge** about thorough cooking for preventing food poisoning as most strongly agreed (46%) or agreed (41%) that eating insufficiently cooked minced meat or raw eggs (e.g., raw dessert mixture) is a risk of food poisoning (Table 1). Other studies also wrote about consumers’ knowledge on consumption of undercooked or raw foods being unsafe, increasing risks of foodborne illness and food poisoning. In Slovenia, consumers knew that improperly prepared raw poultry meat presents a health risk (75%) [37], and elderly consumers knew that chicken meat needs to be cooked sufficiently (98%) in order to prevent food poisoning (71%) [43]. Consumers in China, pre-COVID-19 and post-COVID-19, knew that raw or undercooked seafood (88% and 82%, respectively) and inadequately cooked red meat or chicken (87% and 79%, respectively) presents a risk of foodborne illness [27]. Consumers in the Republic of Korea knew that incompletely cooked meat, fish/shellfish, and eggs are completely hazardous (46% in 2010 and 38% in 2019) and mostly hazardous (44% in 2010 and 42% in 2019) [11]. Consumers knew that consumption of raw or undercooked foods creates risk of food poisoning in Poland (84% meat and 79% eggs) [9], Bosnia and Herzegovina (84% meat and 67% eggs/soft yolks) [8], eight Southeast Asian countries (Brunei, Cambodia, Indonesia, Lao People’s Democratic Republic, Malaysia, the Philippines, Thailand and Vietnam) (84% poultry) [28], Sweden (58% of university students: rare/pink hamburger) [33], Thailand (20% meat and 27% eggs) [9], and Bangladesh (women: 7% meat and eggs and 50% unheated canned food) [14]. In Malaysia, 53% of consumers knew runny eggs are not safe [12], while in Egypt, 36% of consumers knew solid eggs in omelets are safe [13]. Consumers knew that proper cooking prevents food poisoning in Malaysia (95% of postgraduate university students) [29], and Lebanon (77%—meat) [32].

Most CFSS participants had positive **attitudes** about checking whether foods such as meat, poultry and fish are sufficiently heat-treated (roasted/cooked) as this was very important for 57% and important for 37% (Table 2). In Lebanon, 30% of consumers had a positive attitude towards not consuming medium rare chicken (pink on the inside) [30].

Sufficient heat treatment of food (e.g., meat, poultry, and fish) was very important to more than two thirds of CFSS participants (67%) and important to less than a third (29%), indicating very positive **attitudes** towards thoroughly cooking food (Table 2).

CFSS participants had diverse **practices** for assessing whether food is sufficiently cooked when heat-treating meat (including minced meat)/poultry/fish. Participants checked whether food is cooked using a thermometer (12%), by looking at the color of the flesh (61%), by considering the heat-treatment time according to experience or the recipe (68%), and other approaches (8%) (Table 3). These results show the need for improvement in safer food preparation practices, as 1. the recommended method of using a food thermometer is only used by approximately one tenth of participants and 2. each of the two unsafe approaches of relying on color or time is used by roughly two thirds of participants. The use of food thermometers should be better introduced and promoted to consumers. Published reports described different methods used by consumers to determine when food is cooked. Thermometers were used by smaller numbers of consumers in the United Arab Emirates (36% of women) [31], Ireland (26% owned and used—chicken) [15], the U.S. Virgin Islands (20% thermometer or thermometer and subjective method—beef) [19], Egypt (10%—hamburger or chicken) [13], Lebanon (9%—for meat) [32], Sweden (4% of university students—hamburger) [33], and Bosnia and Herzegovina (<1% used it) [8]. In the USA, 20% of parents of elementary school children from Texas owned a food thermometer and 28% of those consistently used it [20], whereas older adults mostly did not use a food thermometer, or only used it for whole poultry (turkey and duck) or for unfamiliar dishes [7]. In Lebanon, 35% of consumers correctly determined if food was sufficiently cooked [26]. Interestingly, in Malaysia, most consumers tended to disagree that a thermometer should be used to check if food is thoroughly cooked [12]. Consumers in Ireland had different opinions regarding the use of thermometers to determine if meat is cooked: 65% were confident with their own method, 5% thought thermometers are only for restaurants, and 3% thought it is a waste of money to buy a thermometer [15]. Subjective methods (e.g., smell, taste, color, etc.) to determine when food is cooked enough were more frequently used by consumers in Egypt (90% unsafe practices—hamburger or chicken) [13], Slovenia (81% of elderly consumers: taste, observation and cooking time) [43], the U.S. Virgin Islands (80%—color, texture, flavor or cooking time—beef meat) [19], Lebanon (65%—smell, taste, color or did not know) [26], Ireland (50% juice runs clear, 32% visual check, 15% duration of cooking, 4% touching it—**chicken** [15], and Malaysia (most consumers: taste or visual appearance e.g., fish opaque and flaky or eggs firm) [12]. In Sweden, 40% of university students cut and checked if the color of hamburger is grey, 13% checked the juice is clear, and 1% relied on frying time of hamburger [33].

#### 3.2.3. Keeping Heat-Treated Food Hot

Most CFSS participants showed good **knowledge** about the importance of keeping heat-treated food warm as 31% strongly agreed or 41% agreed that by keeping heat-treated food warm (at a temperature above 63 °C) before serving we take care of food safety (Table 1). Interestingly, a quite high share of CFSS participants—almost 9% do not know about this and 15% said they neither agree nor disagree (Table 1). Knowledge that hot foods should be kept hot was previously described for consumers in the following countries: Ghana (78% of university students >60 °C) [34], Malaysia (60% >60 °C [12] and 28% >65 °C [35]), Bangladesh (25% >65 °C) [36], Jordan (6% of women: >60 °C) [16], and Egypt (11%—meals are safe at 74 °C) [13]. Varying awareness of the risks of leaving cooked food at room temperature was reported for consumers in Jordan (23% of women knew precooked meals served 3 h later should be refrigerated and warmed when ready to eat) [16], and Slovenia (77% knew microorganisms can multiply in cooked foods left on kitchen counter) [37]. Consumers in Poland (70%) and Thailand (66%) knew that consuming cooked food left at ambient temperature for 12–24 h poses a high risk of food poisoning [9].

CFSS participants had varying **practices** for keeping heat-treated foods (such as soups, meat, side dishes, and sauces) warm until consumption: 18% always do so, 35% almost always, 20% sometimes, 15% mostly not, 7% never, and 2% do not know (Table 4). Although food safety recommendations specify that heat-treated foods (e.g., soups, meat, side dishes, sauces, etc.) should be kept at temperatures above 63 °C until consumption, most CFSS participants do not adhere to this guideline. Only 9% always keep food above 63 °C, 21% almost always, 21% sometimes, 20% mostly not, 15% never, and 12% do not know (Table 4). Most consumers (79%) in Lebanon do not consume food left at room temperature for more than 6 h [10]. In Türkiye, 37% of consumers never and 30% rarely left cooked fish at room temperature until serving [25]. In Serbia, university students had different methods of storing a hot meal for another person to eat several hours later 32–40% used a warm oven, 27–39% the fridge, 23–24% a cool oven, and 10–13% left food on the counter [18].

#### 3.2.4. Leftovers

Before answering the section of the questionnaire about leftover food, the CFSS participants were asked what they usually do with leftover food. The vast majority replied they save leftovers and use them later (92%) and only a few throw leftovers away (8%) (Table 5). This was a filter question and only the participants who store leftovers were answering the following questions about leftover food. In Jordan, 55% of women do not save leftovers for later, but discarded leftover food [16]. Around two thirds of consumers in the UK and Ireland disposed of leftovers or gave them to pets [41].

**Attitudes** of CFSS participants regarding leftovers being cooled down within 2 h after heat treatment and then stored in the refrigerator or freezer were also quite positive as this was very important for 37% and important for 49%, but neither important nor unimportant for 11% (Table 2).

CFSS participants reported varied **practices** for cooling and storing leftover food that has been heat-treated. The majority of CFSS participants (88%) cool the food on the kitchen counter, which is not the safest approach (Table 6). Less than a third (29%) cool the food in smaller portions in the refrigerator and only a tenth (10%) cool the food immediately in cold water (Table 6). It is important that food is cooled down and stored within 2 h. Cooling in cold water helps speed up the cooling. Similarly, small portions cool down faster. So, the main aspect of concern regarding cooling food on the kitchen counter is that food should be cooled down and stored within 2 h. Food safety recommendations to write the name of the food and the date of preparation on the packaging are also not that popular in the practices of CFSS participants. Around one third of participants write the name of the food on the packaging (36%) and slightly less than a third writes the date of preparation on the packaging (31%) (Table 6). To prevent the growth of microbes in favorable temperature conditions it is important to store leftover food in the refrigerator or freezer within 2 h after preparation. Regarding this aspect, 75% of participants have very good practices of putting away cooled food within 2 h after heat treatment (Table 6). Furthermore, the vast majority (97%) store food chilled in the refrigerator/freezer (Table 6).

Consumer practices for storing leftovers varied across countries. Among consumers in Bangladesh, 24% always, 20% often, 24% sometimes, 12% occasionally, and 20% never kept leftover food from lunch at room temperature until dinner [36]. In Sweden, university students mostly stored leftovers in the refrigerator after cooling at room temperature for less than 4 h (69%), actively cooling (15%), leaving at room temperature for 4 h or longer but not the entire day/night (6%) or leaving at room temperature the entire day/night (1%), whereas 9% refrigerated immediately [33]. Consumers in Malaysia mostly tended to avoid keeping leftovers at room temperature until the next meal [12]. In Saudi Arabia, women (73%) stored leftover food in the refrigerator or freezer or kept the food at room temperature until the next meal (10%) [24]. In the UK and Ireland, only about one-third of consumers stored leftovers in the fridge [41], whereas in the U.S. Virgin Islands, 51% refrigerated within 1 h of cooking, and others fed animals (13%), discarded (9%), fed other people (2%), froze (1%), and composted (<1%) [19]. In Lebanon, 60% of consumers did not keep leftovers at room temperature until the next meal/dinner [10]. In the United Arab Emirates, 72% of women immediately refrigerated leftovers [42]. Among student dietitians, 59% cooled down leftovers before refrigeration (88% in the UK, 60% in Lebanon, and 32% in the USA) [38]. Among university students in the USA, 3% did not store leftovers, most stored leftovers in the refrigerator (83%) using covered (85%) or shallow (76%) containers, whereas only 1% kept leftovers at room temperature [39]. Consumers in Poland (54%) and Thailand (43%) ate cooked foods stored at room temperature for longer than 6 h [9]. In Poland, most consumers never or rarely left leftovers in the pot/on the stove/in the oven until eaten (54%), they put still-warm leftovers in the refrigerator (82%), froze leftover meals (85%), or cooled leftovers to room temperature and refrigerated afterwards (50%) [21]. In the Republic of Korea, more consumers stored leftovers in the refrigerator after chilling at room temperature (56% in 2010 and 69% in 2019) than at room temperature (40% in 2010 and 24% in 2019) and even a smaller number refrigerated after chilling in cold water (3% in 2010 and 4% in 2019), or refrigerated immediately (2% in 2010 and 4% in 2019) [11]. In Slovenia, 54% of consumers cooled leftovers at room temperature before refrigerating, 20% fed leftovers to animals, 13% left on the stove, 11% discarded, 1% froze, and 1% refrigerated immediately [22], whereas elementary school children always (29%) and almost always (20%) stored leftovers in the refrigerator [40]. Unsafe practices observed in Italy included leaving boiled milk at room temperature and storing leftovers in improper containers such as aluminum foil for very salty or acidic foods, plastic containers for hot foods, and cookware instead of containers [50].

Before eating leftover food from an already prepared warm meal, the safest approach is to bring the food to the boil or, if that is not applicable (for solid foods), to thoroughly warm it. Two thirds of CFSS participants (67%) thoroughly reheat leftover food to bring it to the boil (Table 5). Less than a third (31%) only reheat leftovers until they are warm enough for immediate consumption (Table 5). A small number of participants (3%) do not reheat leftovers (Table 5). Studies reported diverse approaches to reheating leftovers, differing in terms of whether reheating was performed at all, the methods and locations used (e.g., stove, microwave, or oven), and the extent or duration of heating (from lukewarm to boiling or prolonged boiling). Only small numbers of consumers in Poland (3%) [21] and Slovenia (9% [22] and 11% of elderly [43]) reported not reheating leftovers. In the Republic of Korea, consumers reported reheating food always (46% in 2010 and 51% in 2019), frequently (46% in 2010 and 34% in 2019), sometimes (7% in 2010 and 12% in 2019), seldom (1% in 2010 and 2% in 2019), and never (1% in 2010 and 1% in 2019) [11]. Reheating was practiced by consumers in the USA (university students: 54% depending on type of food, 45% always, or 1% never) [39], the UK and Ireland (around a third) [41], and Bangladesh (26% before consumption) [14].

For reheating of leftovers, university students in the USA [39] and consumers in Slovenia [22] reported using 1. the microwave (96% [39] and 37% [22]), 2. the stove (40% [39] and 46% [22]), or 3. the oven (39% [39] and 6% [22]).

Consumers differed on how long leftovers had to be reheated in the following countries: Ghana (78% of university students: until food was piping hot) [34], Saudi Arabia (women: 66% until it was warm and 29% until it was steaming hot throughout) [24], the United Arab Emirates (50% of women: until boiling) [42], and Egypt (35% until boiling and 65% reported unsafe practices) [13]. In the USA, university students reheated leftovers until food was hot in center (56%), hot to touch (31%), not cold to touch (5%), whereas only 4% used a thermometer [39]. In Poland, consumers reported reheating leftovers until warm to eat (49%), until boiling (31%), and boiling for a few minutes (14%) [21]. Consumers in Slovenia reported reheating leftovers until warm and ready for consumption (42%), until boiling (29%), or boiling for a while (18%) [22], whereas elementary school children always (31%) and almost always (21%) reheated food to boiling [40], and elderly consumers reheated until lukewarm (23%) [43].

Most CFSS participants use up the leftover food stored in the refrigerator within 2 days (81%), others use it within 3 to 4 days (16%) and very few use it after more than 4 days (1%) or do not know (2%) (Table 5). Consumers varied in their practices of how soon they used leftovers. Around a third of consumers in the UK and Ireland used leftovers the same day or within 24 h [41]. University students in the USA kept leftovers for 1–2 days (20%), 3–6 days (43%), a week (31%), two weeks (5%), or longer than two weeks (1%) [39]. During the COVID-19 pandemic consumers in India reported opposite practices related to the consumption of cooked food that was kept in the refrigerator for more than 3–4 days: 27% decreased and 24% increased consumption [44].

The findings of the CFSS revealed several instances of partial compliance, where consumer knowledge does not fully translate into safe practices and behavior. Cooling leftovers on the counter and improper thawing on the counter are often perceived as low-risk, yet these practices keep foods within the danger-zone temperature range, promoting microbial growth during prolonged period of time. The persistent practices of cooling on the counter, despite high awareness of the 2-h rule, illustrated a clear knowledge–practice gap. Reheating practices further showed reliance on subjective approaches, with many consumers boiling food while others reheat only until it feels “warm enough”, raising questions about whether they distinguish between food that is sensed as warm (warm enough) and microbiologically safe. Together, these findings highlight the influence of subjective safety criteria, personal experience, and confidence in one’s own judgment on consumer food-handling decisions.

### 3.3. Structural Equation Modeling (SEM) and the Relationships Between Knowledge (K), Attitudes (A) and Practices (P)—KAP

#### 3.3.1. Structural Equation Modeling (SEM)

**Figure 1:**
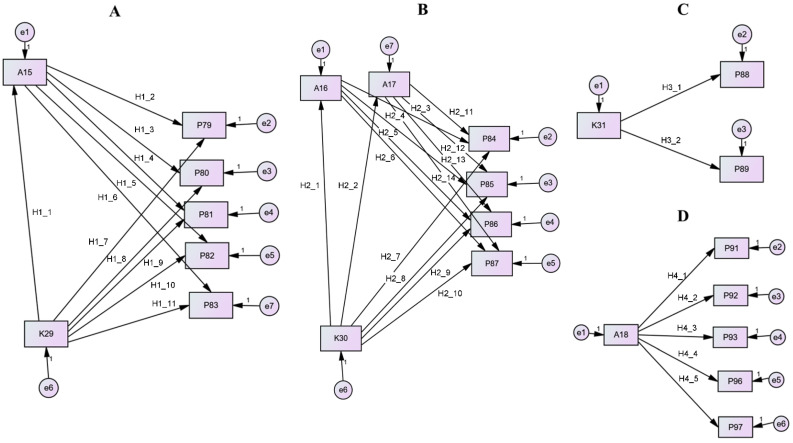
Structural equation models: Model 1 for Thawing (**A**); Model 2 for Thorough cooking (**B**); Model 3 for Keeping hot foods hot (**C**); and Model 4 for Leftovers (**D**). All abbreviations for KAP questions are explained in Table 1, Table 2, Table 3, Table 4, Table 5 and Table 6.

Four structural equation models (SEMs) (Figure 1) were constructed to evaluate the hypothesized pathways linking knowledge (K), attitudes (A), and practices (P) for the topical section 6, “Thawing, heat treatment of food and leftovers”, from the CFSS questionnaire. The models corresponded to four specific food safety topics: Thawing (Model 1), Thorough cooking (Model 2), Keeping hot foods hot (Model 3), and Leftovers (Model 4) (Table 7). Each model was designed to assess topic-specific relationships and to determine how KAP components influenced one another within these domains.

Model 1 examined the effects of knowledge variable K29 on attitudinal factor A15 (hypothesis (H): H1_1) and on practice indicators P79–P83. The model tested both the direct effects of K29 on practices (H1_7–H1_11) and the indirect effects mediated through A15 (H1_2–H1_6). Model 2 assessed the influence of knowledge variable K30 on attitudinal factors A16 and A17 (H2_1, H2_2) and the corresponding practice indicators P84–P87. This model included the direct effects of K30 on practices (H2_7–H2_10) as well as the indirect effects mediated by A16 (H2_3–H2_6) and A17 (H2_11–H2_14). Model 3 investigated the direct effects of knowledge variable K31 on practice indicators P88 (H3_1) and P89 (H3_2). Model 4 explored the direct links between attitudinal factor A18 and practice indicators P91, P92, P93, P96, and P97 (H4_1–H4_5).

Prior to analysis, the data was examined for distributional properties using the Shapiro–Wilk and Anderson–Darling tests. Since these tests indicated departures from normality for Likert-type variables, nonparametric procedures were applied where appropriate. The same dataset was subsequently used for SEM. SEM analyses (Models 1–4) evaluated four key assumptions: multivariate normality, multicollinearity, sample size adequacy, and positive definiteness.

Multivariate normality was assessed using regression diagnostics. No multivariate outliers were identified among the 1621 participants based on the Mahalanobis distance criteria [52]. Multicollinearity was not detected [53], as variance inflation factor (VIF) values remained below the recommended threshold of 10 (range: 1.022–2.990), and tolerance values exceeded 0.01 (range: 0.334–0.979) [54]. Linearity was not explicitly tested due to the ordinal nature of Likert-scale data. However, additional diagnostics indicated no violations of linearity or homoscedasticity. Homogeneity of variance was further supported by comparable variance values across variables (range: 0.233–1.593).

Sample size adequacy was evaluated using an online sample size calculator [55]. The analysis indicated that a minimum of 700 participants was required to achieve a statistical power of 0.80, assuming two latent variables, two observed variables, an anticipated effect size of 0.30, and a significance level of 0.05. Moreover, a minimum sample size of 1621 participants was required to support the specified model structure. This criterion was fully met in the present study.

SEM models were employed to examine the effects of knowledge on attitudes and practices, as well as the influence of attitudes on practices. This approach allows simultaneous estimation of all path coefficients while evaluating the overall model fit [56,57]. The proposed hypotheses and models were tested using structural equation modeling (Figure 1), with a summary of the models and corresponding food safety topics presented in Table 7.

The SEM results for **Model 1** (Table 8), representing Thawing, revealed a clear and theoretically consistent pattern linking knowledge, attitudes, and thawing practices. The latent variable “knowledge that proper thawing of frozen food ensures food safety” (K29) significantly predicted the “attitude towards the way of thawing frozen food” (A15), with a strong and highly significant effect. This indicates that consumers with greater knowledge about the importance of proper thawing for food safety tend to have more positive attitudes regarding appropriate thawing methods. Furthermore, “attitude towards the way of thawing frozen food” (A15) significantly predicted several thawing-related practices: “practice of thawing frozen food on the counter at room temperature” (P79), “practice of thawing frozen food in the microwave” (P80), and “practice of thawing frozen food in cold water” (P81). Interestingly, a significant negative association emerged between “attitude towards the way of thawing frozen food” (A15) and “practice of thawing frozen food in the refrigerator” (P82), suggesting that more permissive or less safety-oriented attitudes may correspond to a lower likelihood of choosing the recommended method of thawing in the refrigerator. The relationship between “attitude towards the way of thawing frozen food” (A15) and “practice of thawing frozen food in other ways” (P83) was not statistically significant. Direct paths from “knowledge that proper thawing of frozen food ensures food safety” (K29) to the thawing practices were mostly non-significant for “practice of thawing frozen food on the counter at room temperature” (P79), “practice of thawing frozen food in the microwave” (P80), “practice of thawing frozen food in cold water” (P81), and “practice of thawing frozen food in the refrigerator” (P82)—indicating that knowledge alone does not translate into thawing practices without the mediating influence of attitudes. The only exception was a small but significant positive effect of “knowledge that proper thawing of frozen food ensures food safety” (K29) on “practice of thawing frozen food in other ways” (P83). Overall, these results show that knowledge about thawing strongly predicts attitudes, but knowledge mostly influences thawing practices indirectly, through attitudes. Consumers with a better understanding of how proper thawing contributes to food safety develop stronger attitudes—favorable or unfavorable—toward various thawing approaches. These attitudes then drive actual thawing practices.

The SEM analysis for **Model 2** (Table 8), covering Thorough cooking, showed that “knowledge that eating insufficiently cooked minced meat or raw eggs (e.g., raw dessert mixture) presents a risk of food poisoning” (K30) significantly predicted both “attitude towards checking whether foods such as meat, poultry and fish, are sufficiently heat-treated (roasted/cooked)” (A16) and “attitude towards sufficiently heat treating food (e.g., meat, poultry, fish)” (A17), as indicated by the strong and highly significant loadings of K30 on A16 and A17. The attitude indicators (A16 and A17) displayed differential predictive effects on cooking assessment practices. “Attitude towards checking whether foods such as meat, poultry and fish, are sufficiently heat-treated (roasted/cooked)” (A16) significantly predicted “practice of assessing whether meat/poultry/fish is already cooked by looking at the color of the flesh” (P85) and “practice of assessing whether meat/poultry/fish is already cooked in other ways” (P87). “Attitude towards sufficiently heat treating food (e.g., meat, poultry, fish)” (A17) significantly predicted “practice of assessing whether meat/poultry/fish is already cooked by considering the heat-treatment time according to experience or the recipe” (P86) and “practice of assessing whether meat/poultry/fish is already cooked in other ways” (P87). Direct paths from “knowledge that eating insufficiently cooked minced meat or raw eggs (e.g., raw dessert mixture) presents a risk of food poisoning” (K30) to the practice indicators were generally weak and mostly non-significant, suggesting that the influence of knowledge on actual cooking practices is largely mediated by attitudes. The only significant direct effect was a negative association with “practice of assessing whether meat/poultry/fish is already cooked in other ways” (P87), suggesting a small but meaningful influence of knowledge on discouraging other assessment strategies. Overall, the findings from Model 2 suggest that knowledge about the risks of undercooked foods primarily influences behavior indirectly through attitudes. Knowledge strongly shapes attitudes toward checking doneness and ensuring sufficient heat treatment, indicating that consumers who understand the food safety risks of undercooked foods tend to hold stronger, more safety-oriented attitudes toward verifying doneness and ensuring adequate cooking. These attitudes influence specific practices—particularly those related to color-based assessment, time-based assessment, and other methods. The direct effects of knowledge on actual cooking behaviors are minimal, highlighting the critical mediating role of attitudes in translating food safety knowledge into real-world heat-treatment practices.

For **Model 3** (Table 8), representing Keeping hot foods hot, the SEM analysis indicated that the latent knowledge variable significantly predicts self-reported practices related to maintaining food temperature. Specifically, the factor “knowledge that by keeping heat-treated food warm (at a temperature above 63 °C) before serving, we take care of food safety” (K31) positively influenced both the “practice of keeping heat-treated food (e.g., soups, meat, side dishes, sauces, etc.) warm until consumption” (P88) and “practice of keeping heat-treated food (e.g., soups, meat, side dishes, sauces, etc.) warm at a temperature above 63 °C until consumption” (P89). Importantly, the association was stronger for maintaining the recommended 63 °C threshold (P89) than for simply keeping food warm in general (P88). This indicated that consumers who knew why keeping heat-treated foods above 63 °C is important for food safety were more likely to keep their foods hot at temperatures above 63 °C in practice. Overall, Model 3 supports a direct connection between knowledge and practices regarding keeping heat-treated foods warm.

The SEM analysis for **Model 4** (Table 8), covering Leftovers, showed that the attitude toward properly cooling and storing leftover food significantly influenced specific food-handling practices. The attitudinal item “attitude towards leftovers cooled down within 2 h after heat treatment and then stored in the refrigerator or freezer” (A18) negatively predicted “practice of cooling leftover heat-treated food on the kitchen counter” (P91) and positively predicted “practice of cooling leftover heat-treated food immediately in cold water” (P92) and “practice of putting away leftover heat-treated food within 2 h after heat treatment” (P96). These relationships indicate that consumers who value timely cooling and proper storage are more likely to adopt these recommended safety practices. However, the influence of “attitude towards leftovers cooled down within 2 h after heat treatment and then stored in the refrigerator or freezer” (A18) did not extend to all cooling or storage behaviors as the remaining two practice indicators “practice of cooling leftover heat-treated food in smaller portions in the refrigerator” (P93) and “practice of storing leftover heat-treated food in the refrigerator/freezer” (P97) were not significantly predicted. These non-significant connections suggest that while attitudes focused on the importance of timely cooling, strongly influence how soon leftovers are cooled and stored, they do not necessarily determine how food is portioned for cooling or whether it is stored appropriately afterward. Overall, Model 4 shows that, regarding Leftovers, the influence of attitude is stronger for some practices than for other practices. Model 4 confirmed that consumers who place high importance on rapid leftover cooling and storage are more likely to 1. prevent prolonged leaving of leftovers in the temperature danger zone, 2. avoid counter cooling and 3. act to store leftovers quickly. However, certain practices related to portioning and general storage of leftovers may depend on other factors beyond the specific attitude investigated in the CFSS.

#### 3.3.2. Relationships Between Knowledge (K), Attitudes (A) and Practices (P)—KAP

In the CFSS, **knowledge** was proven to affect **attitudes** about the topics of Thawing (Model 1) and Thorough cooking (Model 2) (Table 8). The influence of knowledge on attitudes was also previously confirmed for other topics investigated in the CFSS, including Refrigerator temperature, Food labels and expiration dates, and Washing of raw meat and poultry [45], as well as Washing hands and Cleaning the kitchen [46]. The findings from the CFSS regarding food safety knowledge impacting attitudes agree with the findings of studies from the following countries: China and Syria [58], Bangladesh [59], Ghana [60], Sweden [61], Brazil [62], Romania [63], and Türkiye [64]. One study from Malaysia reported that the relationship between knowledge and attitudes was not significant [12].

The influence of **knowledge** on **practices** was fully confirmed only for the topic of Keeping hot foods hot (Model 3: H3_1 and H3_2) (Table 8). Two hypotheses were confirmed for the topics Thawing (Model 1: H1_11) and Thorough cooking (Model 2: H2_10), while the other hypotheses for the topics Thawing (Model 1: H1_7-H1_10) and Thorough cooking (Model 2: H2_7-H2_9) were not confirmed (Table 8). The effect of knowledge on food safety practices was confirmed in the CFSS for several food safety topics including Placement of foods in the refrigerator, Washing of fruits and vegetables, Washing of raw red meat and poultry [45] and Cleaning the kitchen [46], as well as mostly confirmed for the topic of Pets and hygiene [46]. Depending on the topic, the CFSS findings mostly align (H1_11, H3_1, and H3_2) with studies that found there to be a positive impact of knowledge on practices in Bangladesh [59], Romania [63], and Türkiye [64]. A study from Malaysia found that knowledge had a significant negative relationship with food safety behavior (practice) [65]. In contrast, studies from Ghana [60] and Malaysia [12] concluded that knowledge did not have a statistically significant impact on practices. The influence of knowledge on practices [61,64,66] or intention to engage in safe food-handling practices [62] could also be indirect via 1. attitude [61,62,64], 2. perceived behavioral control [62,66], or 3. subjective norms [62].

Findings from the CFSS mostly confirmed the effects of **attitudes** on **practices**. Most hypotheses were confirmed for Thawing (Model 1: H1_2-H1_5) and Leftovers (Model 4: H1_2-H1_5) (Table 8). In contrast, only half of the hypotheses were confirmed for Thorough cooking (Model 2: H2_4, H2_6, H2–13, H2_14) (Table 8). The CFSS study has confirmed the influence of attitudes on practices for the topics of Refrigerator temperature, Food labels and expiration dates [45] and Cleaning the kitchen [46] as well as mostly confirmed for the topics of Washing of raw meat and poultry [45] and Washing hands [46]. These observations from the CFSS indicate that the impact of attitudes on practices might depend on the specific context of the food safety topic. This agrees with a study from China and Syria that reported that the effects of attitudes on practices depend on the topic [58]. Furthermore, evidence from multiple countries confirmed the positive influence of attitudes on practices, as shown in the studies from Bangladesh [59], Sweden [61], Romania [63], Türkiye [64], and Malaysia [12,65]. A study from Ghana reported that the connection between attitudes and practices was not statistically significant [60].

Overall, the CFSS has mostly confirmed the influence of knowledge on attitudes and practices as well as the influence of attitudes on practices. The strength of these connections can depend on the food safety topic and even specific context within the topic. Knowledge was also found to have an indirect effect on practices through attitudes. The relationships between food safety knowledge, attitudes and practices can be complex. The results indicate that attitudes function as a critical mediating mechanism, translating food safety knowledge into behavior, particularly in the thawing and cooking domains. These findings indicate that cognitive awareness alone is insufficient to disrupt entrenched habitual patterns and convenience-oriented behaviors, particularly within routinized food handling at home. The findings of the CFSS highlight the importance of interventions aiming to improve consumer food safety focusing on all three aspects of: knowledge, attitudes and practices.

### 3.4. Suggestions for Future Public Health Campaigns

The findings of the CFSS reveal several opportunities for targeted public health interventions aimed at improving consumer food safety practices. As the use of room-temperature thawing is quite popular, clearer communication is needed on 1. how to do this safely (such as duration, in a bowl of water or something similar, but not directly on the counter) and 2. what safe thawing methods are recommended (such as thawing in the refrigerator). Similarly, inconsistent heat-treatment practices indicate that the campaigns should target two goals: 1. promoting the adoption of low-cost kitchen thermometers for higher-risk foods and informing about the recommended internal temperatures for different foods, and 2. providing practical cues to improve safety for consumers who are reluctant to use thermometers. To achieve more positive consumer food safety attitudes, interventions could also consider personal relevance, social norms, and positive outcome expectations, which, together with an increased public discourse about consumer food safety, could support attitude change. To improve leftover handling, some easy-to-remember rules—perhaps as a mnemonic—about rapid cooling within two hours, storage in closed containers, consumption within 3–4 days and reheating thoroughly. Communication could utilize a variety of channels from food packaging to ads on television, radio, social media and internet sites, as well as billboards, etc. To encourage positive change, the informing of consumers could also be done in collaboration with a well-known and respected public figure who could participate in the campaign as a role model for safe food handling and lead by example, which could also help increase positive attitudes. Collectively, these suggestions can support the development of consumer food safety public health resources that could help improve consumer food safety knowledge, attitudes and practices.

### 3.5. Limitations

The CFSS was based on voluntary convenience sampling with a snowball approach, which may result in self-selection bias and limited representativeness. The high proportion of female participants could also be explained by broader national and societal food-handling patterns. Self-reported data was collected via an online questionnaire, which is the most widely used method for consumer food safety KAP research. This methodology made possible a large sample size (*n* = 1621). Observational data collection was not feasible for such large-scale consumer research. SEM analyses did not include demographic factors. Questions related to leftovers were answered only by participants who store leftover food, which may further limit comparability across subgroups. However, despite these limitations, the large sample size and the comprehensive Matrix of Consumer Food Safety framework allowed a robust assessment of consumer KAP in Slovenia leading to valuable findings in a relatively under-investigated European context.

## 4. Conclusions

The Consumer Food Safety Study found that consumers in Slovenia had mostly good knowledge, positive attitudes and safe practices related to 1. Thawing, 2. Sufficient heat treatment of food, 3. Keeping heat-treated food hot, and 4. Leftovers. There is a need for improvements regarding 1. methods for safe food thawing, 2. using thermometers to check when food is cooked enough, 3. keeping heat-treated food hot at a temperature above 63 °C, 4. methods for safe cooling of leftovers, and 5. writing the name of the food and date when leftovers are stored. Structural equation modeling found that the relationships between knowledge, attitudes, and practices can be complex and depend on the individual food safety topic. The effects of knowledge on attitudes were confirmed for the topics of Thawing and Thorough cooking. The relationship between knowledge and practice was more complex. It was fully confirmed only for the topic of Keeping hot foods hot, but only one hypothesis was confirmed for each of the topics of Thawing and Thorough cooking. The influence of attitudes on practices was mostly confirmed for Thawing and Leftovers, but only partially confirmed for Thorough cooking. Findings showed that knowledge also has an indirect effect on practices through attitudes. It is important that all three aspects of knowledge, attitudes and practices are considered when developing campaigns to improve consumer food safety. The use of topic-specific SEM models provided a more precise understanding of behavioral mechanisms, revealed that effects are not universal across practices (behaviors) or contexts, and showed that interventions need to be tailored to the specific context of the unsafe practices that can lead to foodborne disease. The analytical framework of the questionnaire was designed using a newly developed Matrix of Consumer Food Safety, enabling a comprehensive and multi-layered analysis of the interrelated factors of knowledge, attitudes, and practices that affect consumer food safety. The Consumer Food Safety Study focused on several food safety topics and discovered gaps in consumer food safety knowledge, attitudes and practices. Because knowledge, attitudes, and practices are fundamental components of consumer food safety culture, interventions should not only aim to increase knowledge but also to shift established habits, as knowledge alone is insufficient to change routine consumer behaviors. Future studies can build on this research by conducting cross-regional and longitudinal research. The findings of the Consumer Food Safety Study can also be beneficial for public health institutions in their public outreach activities. The Consumer Food Safety Study not only contributes to a better understanding of consumers in Slovenia, it can also be more widely applied to support the development of consumer-oriented food safety solutions across Central European and other regions.

## Figures and Tables

**Table 1:** Food safety knowledge questions and replies about thawing and heat treatment of food.

Questionnaire Topic	Question Number	Question	Replies					
		Indicate to What Extent You Agree with the Following Statements:	Strongly Agree	Agree	Neither Agree nor Disagree	Disagree	Strongly Disagree	Do Not Know
			Frequency (Percentage)					
Thawing, heat treatment of food and leftovers	K29	Proper thawing of frozen food ensures food safety.	646 (39.9%)	797 (49.2%)	113 (7.0%)	18 (1.1%)	2 (0.1%)	45 (2.8%)
	K30	Eating insufficiently cooked minced meat or raw eggs (e.g., raw dessert mixture) is a risk of food poisoning.	738 (45.5%)	668 (41.2%)	151 (9.3%)	36 (2.2%)	10 (0.6%)	18 (1.1%)
	K31	By keeping heat-treated food warm (at a temperature above 63 °C) before serving, we take care of food safety.	503 (31.0%)	659 (40.7%)	247 (15.2%)	64 (3.9%)	10 (0.6%)	138 (8.5%)

**Table 2:** Food safety attitude questions and replies about importance regarding thawing, heat treatment and leftovers.

Questionnaire Topic	Question Number	Question	Replies					
			Very Important	Important	Neither Important nor Unimportant	Unimportant	Not Important at All	Do Not Know
		How Important It Is to You …	Frequency (Percentage)					
Thawing, heat treatment of food and leftovers	A15	In what way do you thaw frozen food	342 (21.1%)	827 (51.0%)	340 (21.0%)	72 (4.4%)	21 (1.3%)	19 (1.2%)
	A16	To check whether foods such as meat, poultry and fish are sufficiently heat-treated (roasted/cooked)	917 (56.6%)	599 (37.0%)	52 (3.2%)	10 (0.6%)	3 (0.2%)	40 (2.5%)
	A17	That the food (e.g., meat, poultry, fish) has been sufficiently heat-treated.	1081 (66.7%)	477 (29.4%)	28 (1.7%)	6 (0.4%)	1 (0.1%)	28 (1.7%)
	A18	That leftovers are cooled down within 2 h after heat treatment and then stored in the refrigerator or freezer.	597 (36.8%)	786 (48.5%)	179 (11.0%)	26 (1.6%)	6 (0.4%)	27 (1.7%)

**Table 3:** Food-handling practices, questions and replies about thawing and heat treatment.

Questionnaire Topic	Question Number	Question	Replies		
				Yes	No
				Frequency (Percentage)	
Thawing, heat treatment of food and leftovers	P79	How do you thaw frozen food?	On the counter at room temperature	1047 (64.6%)	574 (35.4%)
	P80		In the microwave	416 (25.7%)	1205 (74.3%)
	P81		In cold water	397 (24.5%)	1224 (75.5%)
	P82		In the refrigerator	866 (53.4%)	755 (46.6%)
	P83		Other	93 (5.7%)	1528 (94.3%)
	P84	When heat-treating meat (including minced meat)/poultry/fish, how do you assess whether it is already cooked?	I use a thermometer	199 (12.3%)	1422 (87.7%)
	P85		I look at the color of the flesh	983 (60.6%)	638 (39.4%)
	P86		I consider the heat-treatment time according to experience or the recipe	1102 (68.0%)	519 (32.0%)
	P87		Other	126 (7.8%)	1495 (92.2%)

**Table 4:** Food-handling practices, questions and replies about frequency of keeping heat-treated food warm.

Questionnaire Topic	Question Number	Question	Replies					
			Always	Almost Always	Sometimes	Mostly Not	Never	Do Not Know
			Frequency (Percentage)					
Thawing, heat treatment of food and leftovers	P88	How often do you keep heat-treated food (e.g., soups, meat, side dishes, sauces, etc.) warm until consumption?	286 (17.6%)	566 (34.9%)	325 (20.0%)	247 (15.2%)	106 (6.5%)	33 (2.0%)
	P89	How often do you keep heat-treated food (e.g., soups, meat, side dishes, sauces, etc.) warm at a temperature above 63 °C until consumption?	138 (8.5%)	338 (20.9%)	347 (21.4%)	321 (19.8%)	235 (14.5%)	193 (11.9%)

**Table 5:** Food-handling practices, questions and replies about leftovers.

Questionnaire Topic	Question Number	Question	Replies	Frequency (Percentage)
Thawing, heat treatment of food and leftovers	P90	You have some food left after lunch. What do you usually do?	I save the leftovers and use them later	1494 (92.2%)
			I throw the leftovers away	127 (7.8%)
	P98	What do you do before eating leftover food from an already prepared warm meal?	I do not reheat leftovers	44 (2.9%)
			I only reheat leftovers until they are warm enough for immediate consumption	455 (30.5%)
			I thoroughly reheat leftover food to bring it to the boil	994 (66.5%)
	P99	How long does it take to use up leftover food stored in the refrigerator?	I use it within 2 days	1208 (80.9%)
			I use it within 3 to 4 days	240 (16.1%)
			I use it within more than 4 days	21 (1.4%)
			I do not know	24 (1.6%)

**Table 6:** Food-handling practices, questions and replies about cooling and storing leftovers.

Questionnaire Topic	Question Number	Question	Replies			
				Yes	No	Do Not Know
				Frequency (Percentage)		
Thawing, heat treatment of food and leftovers	P91	How do you cool and store leftover food that has been heat-treated?	I cool the food on the kitchen counter	1314 (88.0%)	171 (11.4%)	8 (0.5%)
	P92		I cool the food immediately in cold water	144 (9.6%)	1329 (89.0%)	20 (1.3%)
	P93		I cool the food in smaller portions in the refrigerator	428 (28.6%)	1047 (70.1%)	18 (1.2%)
	P94		I write the name of the food on the packaging	542 (36.3%)	936 (62.7%)	15 (1.0%)
	P95		I write the date of preparation on the packaging	460 (30.8%)	1018 (68.1%)	15 (1.0%)
	P96		I put away cooled food within 2 h after heat treatment	1117 (74.8%)	260 (17.4%)	116 (7.8%)
	P97		I store it chilled in the refrigerator/freezer	1447 (96.9%)	41 (2.7%)	5 (0.3%)

**Table 7:** Overview of SEM models by food safety topics.

Model Number	Questionnaire Section	Food Safety Topic
1	6. Thawing, heat treatment of food and leftovers	Thawing
2		Thorough cooking
3		Keeping hot foods hot
4		Leftovers

**Table 8:** Parameters of the KAP SEM Models 1–4.

Model	Hypothesis	Path	Estimate	S.E.	C.R.	*p*	Result
1	H1_1	A15<---K29	0.608	0.019	32.502	***	Confirmed
	H1_2	P79<---A15	0.102	0.016	6.320	***	Confirmed
	H1_3	P80<---A15	0.049	0.015	3.310	***	Confirmed
	H1_4	P81<---A15	0.029	0.015	2.000	0.046	Confirmed
	H1_5	P82<---A15	−0.083	0.017	−4.944	***	Confirmed
	H1_6	P83<---A15	0.012	0.008	1.575	0.115	Not confirmed
	H1_7	P79<---K29	−0.016	0.016	−0.997	0.319	Not confirmed
	H1_8	P80<---K29	−0.006	0.014	−0.430	0.667	Not confirmed
	H1_9	P81<---K29	−0.006	0.014	−0.409	0.682	Not confirmed
	H1_10	P82<---K29	−0.025	0.016	−1.547	0.122	Not confirmed
	H1_11	P83<---K29	0.019	0.008	2.464	0.014	Confirmed
2	H2_1	A16<---K30	0.331	0.024	13.630	***	Confirmed
	H2_2	A17<---K30	0.316	0.021	15.043	***	Confirmed
	H2_3	P84<---A16	−0.008	0.009	−0.874	0.382	Not confirmed
	H2_4	P85<---A16	−0.079	0.014	−5.749	***	Confirmed
	H2_5	P86<---A16	−0.013	0.013	−1.024	0.306	Not confirmed
	H2_6	P87<---A16	0.044	0.007	6.120	***	Confirmed
	H2_7	P84<---K30	0.005	0.010	0.460	0.646	Not confirmed
	H2_8	P85<---K30	0.026	0.015	1.732	0.083	Not confirmed
	H2_9	P86<---K30	−0.003	0.014	−0.217	0.829	Not confirmed
	H2_10	P87<---K30	−0.023	0.008	−2.912	0.004	Confirmed
	H2_11	P84<---A17	−0.019	0.011	−1.784	0.074	Not confirmed
	H2_12	P85<---A17	−0.006	0.016	−0.372	0.710	Not confirmed
	H2_13	P86<---A17	−0.069	0.015	−4.552	***	Confirmed
	H2_14	P87<---A17	0.054	0.008	6.382	***	Confirmed
3	H3_1	P88<---K31	0.129	0.026	4.970	***	Confirmed
	H3_2	P89<---K31	0.304	0.027	11.154	***	Confirmed
4	H4_1	P91<---A18	−0.037	0.012	−3.006	0.003	Confirmed
	H4_2	P92<---A18	0.053	0.016	3.232	0.001	Confirmed
	H4_3	P93<---A18	0.021	0.018	1.156	0.248	Not confirmed
	H4_4	P96<---A18	0.192	0.018	10.516	***	Confirmed
	H4_5	P97<---A18	0.009	0.009	0.940	0.347	Not confirmed

S.E.—standard errors; C.R.—critical ratios; *p*—probability value; and *** *p* < 0.001. All abbreviations for KAP questions listed in Path column of this table are explained in Table 1, Table 2, Table 3, Table 4, Table 5 and Table 6.

## Data Availability

The original contributions presented in this study are included in the article; further inquiries can be directed to the corresponding authors.

## Appendix A

**Table A1:** Matrix of Consumer Food Safety (MCFS) covering the questions presented in this article.

Topic	Questions		
	Knowledge (K)	Attitude (A)	Practice (P)
**Thawing, heat treatment of food and leftovers**	**K29** To what extent do you agree that proper thawing of frozen food ensures food safety?	**A15** How important is it for you in what way you thaw frozen food?	How do you thaw frozen food? **P79** On the counter at room temperature **P80** In the microwave **P81** In cold water **P82** In the refrigerator **P83** Other
	**K30** To what extent do you agree that eating insufficiently cooked minced meat or raw eggs (e.g., raw dessert mixture) is a risk of food poisoning?	**A16** How important is it for you to check whether foods such as meat, poultry and fish, have been sufficiently heat-treated (roasted/cooked)?	When heat-treating meat (including minced meat)/poultry/fish, how do you assess whether it is already cooked? **P84** I use a thermometer **P85** I look at the color of the flesh **P86** I consider the heat-treatment time according to experience or the recipe **P87** Other
		**A17** How important is it for you that the food (e.g., meat, poultry, fish) has been sufficiently heat-treated?	
	**K31** To what extent do you agree that by keeping heat-treated food warm (at a temperature above 63 °C) before serving, we take care of food safety?		**P88** How often do you keep heat-treated food (e.g., soups, meat, side dishes, sauces, etc.) warm until consumption?
			**P89** How often do you keep heat-treated food (e.g., soups, meat, side dishes, sauces, etc.) warm at a temperature above 63 °C until consumption?
			**P90** You have some food left after lunch. What do you usually do?
		**A18** How important is it for you that leftovers are cooled down within 2 h after heat treatment and then stored in the refrigerator or freezer.	How do you cool and store leftover food that has been heat-treated? **P91** I cool the food on the kitchen counter **P92** I cool the food immediately in cold water **P93** I cool the food in smaller portions in the refrigerator **P94** I write the name of the food on the packaging **P95** I write the date of preparation on the packaging **P96** I put away cooled food within 2 h after heat treatment **P97** I store it chilled in the refrigerator/freezer
			**P98** What do you do before eating leftover food from an already prepared warm meal?
			**P99** How long does it take to use up leftover food stored in the refrigerator?
